# The potential of CBC-derived ratios (monocyte-to-lymphocyte, neutrophil-to-lymphocyte, and platelet-to-lymphocyte) to predict or diagnose incident TB infection in Tanzanian adolescents

**DOI:** 10.1186/s12879-020-05331-w

**Published:** 2020-08-18

**Authors:** Christiaan A. Rees, Dwan B. Pineros, Maryam Amour, Patricia Munseri, Jamila Said, Albert Magohe, Mecky Matee, Kisali Pallangyo, C. Fordham von Reyn

**Affiliations:** 1 Geisel School of Medicine at Dartmouth, One Rope Ferry Road, Hanover, NH 03755 USA; 2 Present Address: Brigham and Women’s Hospital, 75 Francis St, Boston, MA 02115 USA; 3 Present Address: UCSF Benioff Children’s Hospital Oakland, 747 52nd St, Oakland, CA 94609 USA; 4 Muhimbili University of Health and Allied Sciences, PO Box 65001, Dar es Salaam, Tanzania; 5 Geisel School of Medicine at Dartmouth, Dartmouth-Hitchcock Medical Center, One Medical Center Drive, Lebanon, NH 03756 USA

**Keywords:** *Mycobacterium tuberculosis*, Complete blood count, White blood cells, Red blood cells, Tuberculosis infection, TB vaccine

## Abstract

**Background:**

Ratios of different immune cell populations (i.e.*,* monocyte-to-lymphocyte, neutrophil-to-lymphocyte, and platelet-to-lymphocyte ratios) have been studied as a means of predicting future tuberculosis (TB) disease risk or to assist in the diagnosis of incident TB disease. No studies to-date, however, have evaluated the potential of these ratios to predict or assist in the diagnosis of incident TB *infection* - the first step in the natural history of TB disease.

**Methods:**

In this prospective study, we evaluated the complete blood count (CBC)-derived metrics of monocyte-to-lymphocyte ratio (MLR), neutrophil-to-lymphocyte ratio (NLR), and platelet-to-lymphocyte ratio (PLR) as predictors of future TB infection risk or aids in the diagnosis of TB infection among 145 Tanzanian adolescents enrolled in the DAR-901 vaccine trial, using paired CBCs and interferon-gamma release assays (IGRAs) obtained at 0, 60 and 720 days after study enrollment.

**Results:**

At baseline, there were no significant differences between study participants who remained persistently IGRA negative throughout the study period and those who subsequently converted to IGRA positive with respect to MLR (0.18 vs 0.17, *p* = 0.10), NLR (0.88 vs 1.02, *p* = 0.08), or PLR (115 vs 120, *p* = 0.28). Similarly, no significant differences were noted with respect to MLR, NLR, and PLR between IGRA converters and time-matched negative controls at the time of IGRA conversion. With respect to other blood cell measures, however, there were modest but significant differences between IGRA negatives and IGRA converters with respect to red blood cell count (4.8 vs 4.6 ×  10^6^ cells/mcL, *p* = 0.008), hemoglobin (12.6 vs 12.3 g/dL, *p* = 0.01), and hematocrit (38.8 vs 37.8%, *p* = 0.005).

**Conclusions:**

In contrast to prior studies that have suggested that the ratios of different immune cell populations are associated with development of TB disease, our present findings do not demonstrate an association between these ratios and the development of TB infection. However, decreased red blood cell measures were associated with the subsequent development of TB infection, suggesting either that dysregulation of iron metabolism may play a role in TB pathogenesis or that following TB infection, iron dysregulation may precede IGRA positivity.

**Trial registration:**

Clinicaltrials.gov NCT02712424. Date of registration: March 14, 2016.

## Background

The World Health Organization (WHO) estimates that in 2018, there were approximately 10 million incident cases of tuberculosis (TB) resulting in 1.5 million deaths, of which approximately 1.1 million cases and 205,000 deaths occurred in children [1]. TB remains the leading cause of death due to an infectious agent worldwide. Efforts to control TB include the development of laboratory tests capable of identifying those with asymptomatic TB infection who are at risk for subsequently developing TB disease. Examples of such a test include interferon gamma release assays (IGRAs) [2] and genomic signatures [3, 4], although both remain imperfect diagnostic tools. Some studies have proposed the use of more basic laboratory measures, such as the ratios of different immune cell populations in peripheral blood [5–7], for predicting risk of subsequent TB disease development and/or identifying incident TB disease cases. For example, a number of studies have evaluated the monocyte-to-lymphocyte ratio (MLR) in peripheral blood for the purpose of either predicting those at risk of subsequent active TB disease or comparing values in those with active disease versus controls [5–12].

To the best of our knowledge, no prospective studies have evaluated baseline MLR or other complete blood count (CBC)-derived measures such as neutrophil-to-lymphocyte ratio (NLR) or platelet-to-lymphocyte ratio (PLR) to identify those at risk of developing subsequent incident TB infection, the earliest stage in the natural history of tuberculosis. Furthermore, no studies have evaluated CBC components at the time of incident TB infection. Here, we assessed the relationship between TB infection, as defined by IGRA positivity, and concurrent CBC values and CBC-derived ratios (MLR, NLR, and PLR) in a recent TB vaccine trial in Tanzania.

DAR-PIAT was a Phase 2b Prevention of Infection (POI) trial of the investigational TB vaccine DAR-901 [13]. DAR-901 represents a scalable manufacturing process for SRL172, an inactivated whole cell non-tuberculous mycobacterial vaccine shown previously to prevent tuberculosis in a Phase 3 trial [14]. In the present POI trial, adolescents without prior TB infection were enrolled, randomized to vaccine or placebo, and followed 3 years for development of incident TB infection, as defined by conversion from IGRA-negative to IGRA-positive. CBCs were performed at baseline and at follow-up visits. The prospective trial provided a unique opportunity to evaluate the potential utility of baseline MLR, NLR, and PLR, as well as other CBC components, for predicting subsequent development of incident TB infection in an IGRA-negative population, and also for comparing CBC values between TB-infected individuals at the of time of incident infection with time-matched controls.

The CBC ratios described here are hypothesized to represent markers of non-specific inflammation, and have been evaluated in the context of TB disease [5–12, 15–25], malignancy [26–30], and several other conditions that are hypothesized to have a pro-inflammatory component [31–33]. Through the analysis of paired CBC and IGRA assays, we are able to report on the utility of baseline MLR, NLR, and PLR, as well as other CBC components, for predicting future development of asymptomatic TB infection and on the effect of incident TB infection on concurrent CBC values.

## Methods

### Study population and trial procedures

Data included in this study were derived from the prospective DAR-PIAT Phase 2b clinical trial (ClinicalTrials.gov Identifier: NCT02712424) aimed at determining the efficacy of a three-injection booster series of DAR-901 or saline placebo in preventing TB infection among Tanzanian adolescents (ages: 13–15) who had been previously immunized with BCG. This study was approved by the Committee for the Protection of Human Subjects at Dartmouth College. Written assent was obtained from all study participants, and consent obtained from their parent(s) or guardian(s). Adolescents were screened by the T-spot® IGRA for evidence of TB infection. Participants who were IGRA-positive at screening were excluded from participation in the immunization phase of the trial and were referred for further medical evaluation. IGRA-negative adolescents who met study criteria were enrolled in the immunization phase of the trial. Vaccine doses were administered at: day 0 (visit 1), day 60 (visit 2), and day 120 (visit 3).

### Collection and analysis of IGRA and CBC specimens

IGRA testing was performed at: day 0 (visit 1), day 60 (visit 2), day 420 (visit 4), and day 720 (visit 5). Data from the final follow-up visit at 3 years (visit 6) are not included in the present analysis. For participants who were IGRA-positive at day 720, one additional IGRA was obtained after the conclusion of the regular study period. CBCs with white blood cell differentials were obtained using an Abbott Cell-Dyn 3700 Hematology Analyzer (Abbott Laboratories, Chicago, IL) at: day 0 (visit 1), day 60 (visit 2), day 90 (visit 3.7), and day 720 (visit 5). The instrument was operated in accordance with the manufacturer’s instructions. Quality control samples were analyzed twice daily, and instrumental maintenance and calibration were performed according to the timelines specified by the manufacturer. In summary, paired IGRA and CBCs were obtained at day 0 (visit 1), day 60 (visit 2) and day 720 (visit 5).

### Definitions for IGRA converter groups

For the purpose of this study, *IGRA converters* (indicating incident TB infection) were defined as participants who were IGRA-negative at baseline but subsequently converted to IGRA-positive at days 60, 420, and/or 720. Participants who converted to IGRA-positive during the vaccine dosing period (day 60) were defined as *early converters,* while those who converted to IGRA-positive after completion of the vaccine dosing period (either day 420 or 720) were defined as *late converters*. All late converters (both day 420 and 720) were included in the analysis of baseline CBC values. However, those who were IGRA-positive at day 420 but had reverted to IGRA-negative at day 720 were excluded from the analysis at time of IGRA positivity since a concurrent CBC was not collected at day 420. Those who were IGRA-positive at both day 60 and 720 were classified as early converters. *Persistent converters* were defined as participants with two or more positive IGRA results over the study period. *Transient converters* were defined as converters with a single positive IGRA followed by subsequent borderline and/or negative IGRAs. *IGRA-negative controls* were defined as participants who were IGRA-negative at baseline and remained IGRA-negative throughout the entire follow-up period of the study. *Baseline CBC values* were defined as those obtained at the time of enrollment and prior to administration of any study treatment. *Concurrent CBC values* were defined as those obtained at the same visit as the detection of IGRA conversion, noting that conversion could have occurred any time since the prior negative IGRA (range 60–720 days).

### Statistical analyses

The absolute monocyte count divided by the absolute lymphocyte count yielded the MLR, the absolute neutrophil count divided by the absolute lymphocyte count yielded the NLR, and the absolute platelet count divided by the absolute lymphocyte count yielded the PLR. All other laboratory values were obtained directly from CBC results.

The non-parametric Mann-Whitney U-test was used for the comparison of continuous variables, and the Fisher’s exact test for the comparison of categorical variables. Mean, standard deviation, and interquartile ranges are reported where appropriate. Receiver operating characteristic (ROC) curves were generated to evaluate the diagnostic utility of MLR, NLR, and PLR, as well as various CBC components for differentiating between IGRA converters and IGRA-negative participants. Area under the receiver operating characteristic curve (AUROC) was used as a measure of test performance, and Youden’s J statistic was used to identify the cutoff value for each variable that optimized the sum of sensitivity and specificity [34]. All statistical analyses were performed using R version 3.5.0, with the ‘pROC’ package used to generate ROC curves and associated data.

## Results

### Study participant characteristics

A total of 936 adolescents (age: 13–15 years) were screened for evidence of TB infection: 164 were IGRA-positive or borderline at baseline and were excluded from immunization; 667 were IGRA-negative and eligible for the immunization phase of the trial. A total of 48 participants converted to IGRA-positive at day 60, 420, or 720, including 20 early converters (initial positive at day 60, 11 females/9 males) and 29 late converters (initial positive at day 420 or 720, 17 females/12 males). One female early converter was excluded because a paired CBC sample was not collected at the time of IGRA conversion. All other participants who converted to IGRA-positive at day 60, 420, or 720 are included in the present study. Among the IGRA-positive participants, 22 were persistent positives and 25 were transient positives. Twenty-one received the DAR-901 vaccine (12 persistent/9 transient), while 26 received saline placebo (10 persistent/16 transient). Ninety-eight randomly selected participants who were IGRA-negative at baseline and remained IGRA-negative throughout the entire trial period (56 female/42 male) served as negative controls. Of these, 47 received the DAR-901 vaccine, and 51 received saline placebo. The IGRA converters and IGRA-negative controls were similar with respect to both gender (*p* = 1.00) and treatment assignment (i.e.*,* placebo vs vaccine, *p* = 0.73).

### Baseline CBC values of subsequent IGRA converters and IGRA-negative participants

We first evaluated whether baseline CBC values (obtained at screening) differed between subsequent IGRA converters (*n* = 47) and IGRA-negative controls (*n* = 98). At baseline, subsequent IGRA converters had modest but statistically significant reductions in red blood cell (RBC) count (4.6 vs 4.8 × 10^6^/mcL, *p* = 0.008), hemoglobin (12.3 vs 12.6 g/dL, *p* = 0.01), and hematocrit (37.8 vs 38.8%, *p* = 0.004), compared with IGRA-negative controls (Table 1). None of the WBC-associated measures differed between IGRA converters and controls. Among IGRA converter sub-groups, persistent converters had the greatest number of CBC components that were statistically different from IGRA-negative controls (*n* = 4: absolute neutrophil count, RBC count, hemoglobin, and hematocrit), followed by late converters (*n* = 3: RBC count, hemoglobin, and hematocrit), transient converters (*n* = 1: hematocrit), and early converters (*n* = 1: hematocrit).

**Table 1:** Baseline CBC results for subsequent IGRA converters and IGRA-negative participants

g	IGRA-Negative (***n*** = 98)	All Converters (***n*** = 47)		Early Converters (***n*** = 18)		Late Converters (***n*** = 29)		Transient Converters (***n*** = 25)		Persistent Converters (***n*** = 22)	
Laboratory Component	Mean (S.D.)	Mean (S.D.)	***p***-val	Mean (S.D.)	***p***-val	Mean (S.D.)	***p***-val	Mean (S.D.)	***p***-val	Mean (S.D.)	***p***-val
**White blood cells (WBCs)**											
WBC count (× 103/mcL)	6.14 (1.36)	5.81 (1.36)	0.20	5.79 (1.22)	0.46	5.82 (1.47)	0.24	5.79 (1.43)	0.36	5.83 (1.32)	0.29
1Neutrophil count (× 103/mcL)	2.66 (0.96)	2.26 (0.93)	0.06	2.34 (0.94)	0.29	2.22 (0.94)	0.08	2.37 (1.00)	0.36	2.15 (0.85)	**.05**
Neutrophil %	42.5 (8.6)	38.2 (11.8)	0.11	39.6 (11.2)	0.41	37.4 (12.2)	0.12	39.4 (11.1)	0.48	36.8 (12.6)	0.07
Lymphocyte count (× 103/mcL)	2.68 (0.56)	2.73 (0.74)	0.91	2.66 (0.63)	0.94	2.78 (0.81)	0.92	2.66 (0.61)	0.96	2.82 (0.87)	0.90
Lymphocyte %	44.5 (7.6)	47.8 (10.0)	0.12	46.5 (9.2)	0.51	48.6 (10.5)	0.11	47.2 (9.6)	0.39	48.5 (10.5)	0.11
Monocyte count (× 103/mcL)	0.48 (0.151)	0.459 (0.175)	0.40	0.467 (0.153)	0.99	0.454 (0.189)	0.26	0.425 (0.147)	0.10	0.498 (0.197)	0.68
Monocyte %	7.9 (2.0)	7.9 (2.5)	0.61	8.0 (2.2)	0.92	7.8 (2.7)	0.44	7.3 (1.6)	0.18	8.5 (3.1)	0.52
Eosinophil count (× 103/mcL)	0.220 (0.259)	0.253 (0.229)	0.26	0.230 (0.222)	0.88	0.268 (0.236)	0.16	0.249 (0.197)	0.17	0.258 (0.266)	0.75
Eosinophil %	3.5 (3.6)	4.4 (3.9)	0.17	4.2 (4.4)	0.80	4.5 (3.6)	0.09	4.5 (4.0)	0.13	4.3 (3.8)	0.58
Basophil count (× 103/mcL)	0.100 (0.075)	0.097 (0.068)	0.98	0.093 (0.069)	0.66	0.100 (0.069)	0.77	0.084 (0.036)	0.90	0.112 (0.091)	0.93
Basophil %	1.6 (1.1)	1.7 (1.2)	0.60	1.7 (1.4)	0.99	1.7 (1.2)	0.46	1.5 (0.8)	0.69	1.9 (1.6)	0.68
**Red blood cells (RBCs)**											
1,2RBC count (× 106/mcL)	4.8 (0.4)	4.6 (0.5)	**0.008**	4.7 (0.8)	0.20	4.5 (0.4)	**0.008**	4.7 (0.6)	0.08	4.5 (0.4)	**0.02**
1,2Hemoglobin (g/dL)	12.6 (1.0)	12.3 (1.6)	**0.01**	12.5 (2.4)	0.08	12.1 (0.9)	**0.03**	12.4 (2.0)	0.09	12.1 (1.0)	**0.02**
1,2,3,4Hematocrit (%)	38.8 (3.0)	37.8 (4.5)	**0.005**	38.2 (6.5)	**0.03**	37.5 (2.7)	**0.03**	38.1 (5.6)	**0.04**	37.4 (3.0)	**0.02**
MCV (fL)	81.9 (6.2)	82.2 (5.7)	0.67	81.4 (6.7)	0.90	82.7 (5.1)	0.50	81.4 (5.2)	0.68	83.1 (6.3)	0.26
MCH (pg)	26.5 (2.2)	26.8 (2.1)	0.50	26.7 (2.5)	0.58	26.8 (1.9)	0.62	26.6 (2.1)	0.96	27.0 (2.2)	0.30
MCHC (g/dL)	32.4 (0.9)	32.5 (0.9)	0.36	32.8 (0.8)	0.12	32.4 (1.9)	0.94	32.6 (0.9)	0.31	32.5 (1.0)	0.71
RDW (%)	16.1 (1.5)	16.7 (2.4)	0.33	16.9 (2.9)	0.38	16.6 (2.2)	0.51	17.2 (3.0)	0.15	16.1 (1.5)	0.99
**Platelets**											
Platelet count (× 103/mcL)	312 (103)	30 (118)	0.20	306 (141)	0.24	297 (104)	0.38	288 (107)	0.19	315 (131)	0.51

*S.D.* standard deviation, *MCV* mean corpuscular volume, *MCH* mean corpuscular hemoglobin, *MCHC* mean corpuscular hemoglobin concentration, *RDW* red cell distribution width

### Baseline CBC-derived ratios of subsequent IGRA converters and IGRA-negative participants

Next, we sought to determine whether any of the baseline CBC-derived ratios (MLR, NLR, and PLR) differed between subsequent IGRA converters and IGRA-negative controls. There were no significant differences between all IGRA converters and controls with respect to MLR (0.17 vs 0.18, *p* = 0.10), NLR (0.88 vs 1.02, *p* = 0.08), or PLR (115 vs 120, *p* = 0.28) (Table 2). With respect to sub-group analysis, there was a modest but statistically significant decrease in MLR among transient converters relative to controls (0.16 vs 0.18, *p* = 0.03). No differences were observed in sub-group analyses for either NLR or PLR.

**Table 2:** Baseline MLR, NLR, and PLR for subsequent IGRA converters and IGRA-negative participants

Group	***n***	Mean	S.D.	Q1	Q3	***p-value***
**MLR**						
IGRA Negatives	98	0.18	0.05	0.15	0.21	*Reference*
All Converters	47	0.17	0.06	0.13	0.20	0.10
Early Converters	18	0.18	0.07	0.13	0.22	0.56
Late Converters	29	0.16	0.06	0.13	0.18	0.07
Transient Converters	25	0.16	0.04	0.13	0.18	**0.03**
Persistent Converters	22	0.18	0.08	0.14	0.22	0.73
**NLR**						
IGRA Negatives	98	1.02	0.40	0.74	1.22	*Reference*
All Converters	47	0.88	0.42	0.56	1.21	0.08
Early Converters	18	0.93	0.42	0.62	1.25	0.35
Late Converters	29	0.85	0.42	0.50	1.20	0.09
Transient Converters	25	0.91	0.39	0.68	1.69	0.38
Persistent Converters	22	0.85	0.45	0.48	1.10	0.06
**PLR**						
IGRA Negatives	98	120	46	96	135	*Reference*
All Converters	47	115	50	82	152	0.28
Early Converters	18	119	58	89	136	0.41
Late Converters	29	113	45	80	158	0.40
Transient Converters	25	112	43	79	155	0.22
Persistent Converters	22	119	57	87	142	0.69

*S.D.* standard deviation, *Q*_*1*_ first quartile (25th percentile), *Q*_*3*_ third quartile (75th percentile)

**Fig. 1:**
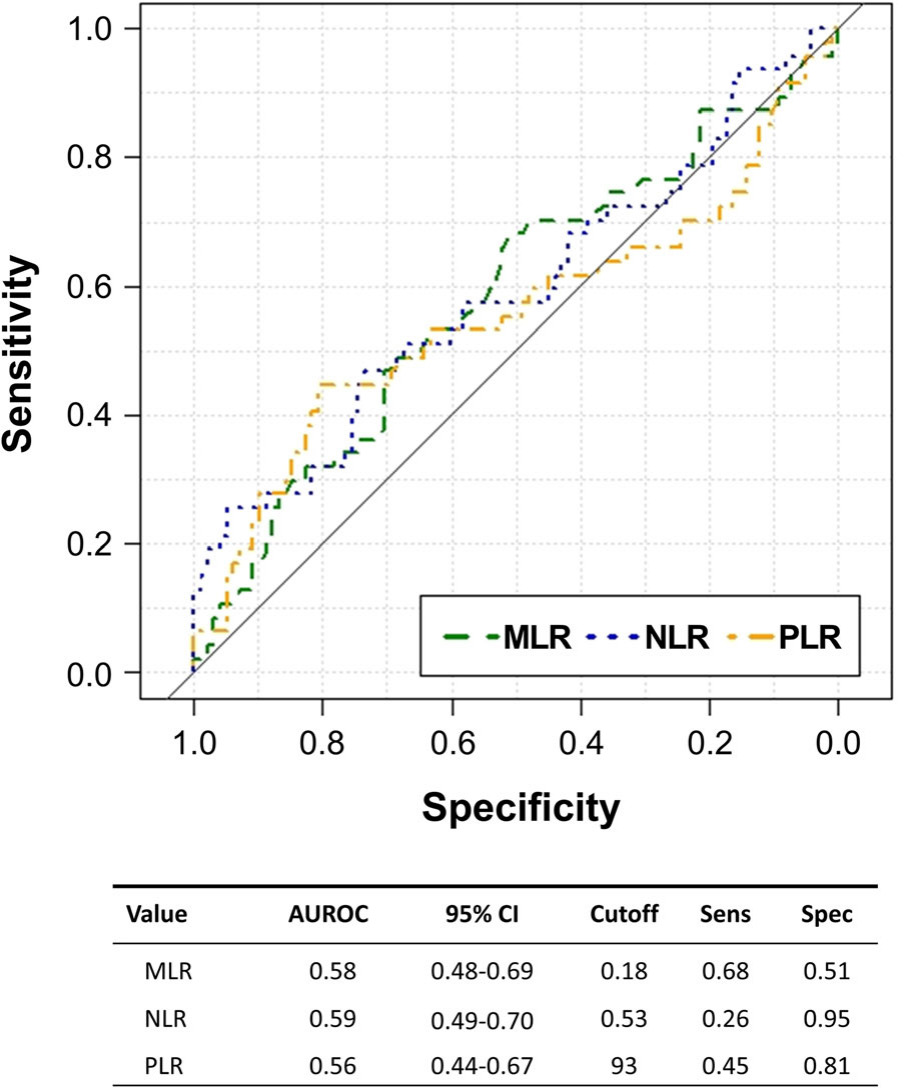
ROC curves for baseline MLR (green), NLR (blue), and PLR (orange) in the comparison of IGRA converters and IGRA-negative participants. AUROC: area under the receiver operating characteristic curve; 95% CI: 95% confidence interval for AUROC; Sens: sensitivity; Spec: specificity

The performance of baseline MLR, NLR, and PLR with respect to identifying subsequent TB infection was evaluated using ROC curves, with the AUROC used to quantify test performance, and Youden’s index used to define optimal cutoff values. With respect to all converters, MLR yielded an AUROC of 0.58 (95% CI: 0.48–0.69), NLR an AUROC of 0.59 (95% CI: 0.49–0.70), and PLR an AUROC of 0.56 (95% CI: 0.44–0.67), with none of these ratios performing statistically better than random (Fig. 1). Of note, RBC count (AUROC: 0.64, 95% CI: 0.54–0.73), hemoglobin (AUROC: 0.63, 95% CI: 0.53–0.73), and hematocrit (AUROC: 0.65, 95% CI: 0.55–0.75) did perform statistically better than random for differentiating between overall IGRA converters and controls. With respect to analysis of IGRA converter sub-groups, MLR performed better than random among transient converters only (AUROC: 0.64, 95% CI: 0.52–0.76), and neither NLR nor PLR performed better than random among IGRA converter sub-groups.

### Concurrent CBC values at the time of IGRA conversion

We next sought to determine whether concurrent CBC values differed between IGRA converters at the time of IGRA conversion (*n* = 18 early converters and 25 late converters) and time-matched IGRA-negative controls (*n* = 93 at day 60 and *n* = 95 at day 720). Follow-up CBCs were not available for a subset of IGRA-negative controls (*n* = 5 at day 60 and *n* = 3 at day 720) and were excluded from these analyses accordingly. Although included in the baseline analysis, participants who had converted to IGRA-positive at day 420 but had reverted to IGRA-negative by day 720 are excluded from the concurrent analysis since a CBC was not obtained at the time of IGRA conversion. Comparison groups were similar with respect to treatment assignment (i.e.*,* vaccine vs placebo) and gender for early converters versus time-matched negative controls (*p* = 0.57 for treatment, *p* = 0.44 for gender), late converters versus time-matched controls (*p* = 0.63 for treatment, *p* = 1.00 for gender), and early converters versus late converters (*p* = 0.76 for treatment, *p* = 0.76 for gender). There were no significant differences noted with respect to any CBC components or CBC-derived ratios (MLR, NLR, or PLR) for either early converters or late converters relative to time-matched controls. In the comparison of early versus late converters, there were significant differences observed with respect to both hemoglobin (12.1 vs 13.0 g/dL, *p* = 0.04) and hematocrit (37.7 vs 40.7%, *p* = 0.05), although these differences were also noted in the comparison of day 60 and day 720 IGRA-negative controls (Table 3).

**Table 3:** Concurrent CBC results and CBC-derived ratios for early and late converters at first study visit where IGRA conversion was detected versus time-matched controls

	Early Converters	Negatives at Day 60	Late Converters	Negatives at Day 720	***p***-value	***p-***value	***p***-value
Laboratory Component	Mean (S.D.)	Mean (S.D.)	Mean (S.D.)	Mean (S.D.)	(EC N60)	LC vs N720)	(EC vs LC)
**White blood cells (WBCs)**							
WBC count1	5.62 (1.18)	5.52 (1.44)	5.86 (1.48)	5.43 (1.42)	0.53	0.13	0.48
Neutrophil count1	2.16 (0.85)	2.27 (1.12)	2.62 (1.24)	2.28 (1.02)	0.92	0.24	0.31
Neutrophil %	37.5 (11.1)	39.3 (10.7)	43.2 (12.6)	41.2 (12.0)	0.57	0.75	0.24
Lymphocyte count1	2.69 (0.54)	2.53 (0.63)	2.53 (0.71)	2.41 (0.71)	0.33	0.45	0.40
Lymphocyte %	48.8 (7.6)	47.0 (9.4)	44.4 (11.5)	45.0 (9.6)	0.50	0.81	0.15
Monocyte count1	0.405 (0.147)	0.428 (0.204)	0.374 (0.158)	0.448 (0.365)	0.77	0.33	0.42
Monocyte %	7.2 (2.1)	7.9 (3.9)	6.5 (2.5)	8.4 (7.0)	0.64	0.09	0.30
Eosinophil count1	0.185 (0.205)	0.177 (0.209)	0.269 (0.281)	0.163 (0.224)	0.84	0.13	0.28
Eosinophil %	3.3 (3.7)	3.1 (3.3)	4.6 (4.8)	3.0 (3.5)	0.66	0.17	0.29
Basophil count1	0.181 (0.292)	0.123 (0.116)	0.071 (0.040)	0.143 (0.201)	0.62	0.46	0.42
Basophil %	3.2 (4.7)	2.4 (2.6)	1.3 (0.9)	2.6 (3.4)	0.55	0.07	0.24
**Red blood cells (RBCs)**							
RBC count2	4.6 (0.6)	4.7 (0.5)	4.7 (0.5)	4.8 (0.5)	0.53	0.68	0.44
Hemoglobin (g/dL)	12.1 (1.4)	12.3 (1.0)	13.0 (1.4)	12.7 (1.3)	0.29	0.42	**0.04**
Hematocrit (%)	37.7 (4.5)	38.5 (3.3)	40.8 (4.8)	40.1 (4.1)	0.22	0.50	**0.05**
MCV (fL)	82.3 (6.1)	82.7 (6.7)	86.0 (4.7)	84.0 (7.0)	0.90	0.27	**0.04**
MCH (pg)	26.5 (2.2)	26.6 (2.8)	27.4 (1.8)	26.7 (2.5)	0.98	0.17	0.14
MCHC (g/dL)	32.2 (1.0)	32.1 (2.1)	31.9 (1.5)	31.8 (1.4)	0.78	0.35	0.68
RDW (%)	17.1 (2.1)	16.6 (1.5)	16.4 (1.6)	16.8 (2.1)	0.57	0.64	0.38
**Platelets**							
Platelet count1	276 (81)	311 (168)	272 (95)	289 (79)	0.32	0.37	0.93
**CBC-derived Ratios**							
MLR	0.15 (0.05)	0.18 (0.10)	0.16 (0.07)	0.20 (0.25)	0.54	0.20	0.88
NLR	0.82 (0.38)	0.94 (0.57)	1.13 (0.69)	1.01 (0.54)	0.45	0.71	0.15
PLR	105 (32)	138 (132)	114 (46)	128 (43)	0.11	0.21	0.53

*EC* early converters, *N*_*60*_ IGRA-negative at day 60 (time-matched controls for early converters), *LC* late converters, *N*_*720*_ IGRA-negative at day 720 (time-matched controls for late converters), *MCV* mean corpuscular volume, *MCH* mean corpuscular hemoglobin, *MCHC* mean corpuscular hemoglobin concentration, *RDW* red cell distribution width; ^1^: units are × 10^3^/mcL; ^2^: units are × 10^6^/mcL

We also determined whether CBC components and/or CBC-derived ratios differed between persistent and transient IGRA converter sub-groups. The persistent (*n* = 22) and transient (*n* = 21) converter groups were similar with respect to treatment (*p* = 0.12), gender (*p* = 0.55), and early versus late converter status (*p* = 0.76). With respect to CBC components, only absolute monocyte count was significantly different between persistent and transient converters (2.06 vs 2.81 ×  10^3^/mcL, respectively, *p* = 0.04). Furthermore, none of the CBC-derived ratios differed significantly between the two groups.

### Effect of vaccination on CBC components and CBC-derived ratios

Finally, we sought to evaluate whether vaccination with DAR-901 had an effect on CBC components or CBC-derived ratios relative to treatment with saline placebo. When including all participants irrespective of disease status, there were no significant differences with respect to CBC components or CBC-derived ratios between participants who received DAR-901 versus saline placebo at day 60 and/or 720. When groups were stratified according to disease status (IGRA-positive versus IGRA-negative), there was a modest but significant difference in RDW between late converters who received vaccine versus those who received placebo (15.6 vs 17.0%, respectively, *p* = 0.03).

## Discussion

In this prospective study, we sought to determine whether previously-described abnormalities in CBC-derived ratios for patients with new *symptomatic* active TB could be detected at the earlier stage of new *asymptomatic* TB infection, as identified by IGRA conversion. Among Tanzanian adolescents enrolled in the DAR-PIAT trial, baseline RBC measures (specifically RBC count, hemoglobin, and hematocrit) were modestly but significantly decreased among subsequent IGRA converters relative to those who remained IGRA-negative throughout the study period. Of note, no significant differences were observed between IGRA converters and controls with respect to WBC measures, platelet count, or CBC-derived ratios. Analysis of IGRA-converter subgroups demonstrated that persistent converters had the greatest number of CBC components that were statistically different from controls at baseline, followed by late converters, transient converters, and early converters. Hematocrit was the only CBC component to be significantly reduced among all IGRA converter sub-groups evaluated. With respect to CBC-derived ratios, MLR was modestly but significantly decreased in transient converters relative to controls, while NLR and PLR were not significantly different in any sub-group analyses.

CBCs collected at the time of IGRA conversion revealed little in the way of differences between IGRA converters and time-matched controls. Specifically, there were no significant differences observed with respect to any CBC components or CBC-derived ratios between either early converters or late converters and time-matched controls, while in the comparison of transient and persistent converters, only absolute monocyte count differed between groups (decreased in the persistent converter group). Notably, in the comparison of early converters versus late converters, late converters had a significantly increased hemoglobin and hematocrit, although a similar time-dependent trend was observed among IGRA-negative controls as well. Finally, with respect to the effect of treatment on CBC components or CBC-derived ratios, no significant differences were observed among participants (including both IGRA converters and controls) who received vaccine versus placebo. In sub-group analysis, however, a modest but significant difference in RDW was observed between late converters who received the DAR-901 vaccine versus those who received saline placebo.

A number of prior studies have evaluated CBC-derived ratios (MLR, NLR, and/or PLR) in the context of symptomatic TB disease, specifically related to either predicting subsequent TB disease development, diagnosing TB disease, or monitoring disease response to antimicrobial therapy [5–12, 15–25]. These studies were heterogeneous with respect to the geographic region from which participants originated, presence of comorbidities (such as HIV), age, concurrent use of TB therapy, and choice of control groups, making comparisons challenging.

MLR is arguably the best studied of the CBC-derived ratios evaluated here, with prior studies demonstrating that: 1) MLR differs between TB cases and controls at the time of symptomatic disease [8–12], 2) MLR normalizes in response to TB therapy [8, 10, 12], and 3) baseline differences in MLR predict subsequent development of TB disease [5–7]. Both increased and decreased MLR values appear to be associated with risk of subsequent TB development [7] or current TB disease [10, 11], although the biological explanation for this phenomenon is unclear. Similarly, NLR has previously been associated with the subsequent development of TB disease [15] and the presence of active TB disease (including both pulmonary and extrapulmonary sites) [18–22, 25], as well as the need for TB retreatment in previously-treated individuals [16], and development of acute respiratory distress syndrome (ARDS) in subjects with miliary tuberculosis [17]. Most prior studies have reported that concurrent NLR is elevated in TB disease subjects relative to controls, but that NLR may be even further elevated in the setting of other acute respiratory infections [24]. Finally, an elevated PLR has been associated with symptomatic TB disease at both pulmonary and extrapulmonary sites [22, 23, 25].

To the best of our knowledge, the present study is the first to evaluate CBC ratios associated with asymptomatic TB infection. Baseline MLR, NLR, and PLR did not differ significantly between subsequent IGRA converters and IGRA-negative controls, although sub-group analysis revealed a modest but significant decrease in MLR among transient converters relative to controls. We suspect that the discrepancy between our findings and those of prior studies that have evaluated CBC-derived ratios in the setting of TB disease likely results from the evaluation of immunologically distinct points in the natural history of tuberculosis (i.e.*,* asymptomatic TB infection versus active TB disease). For example, an elevated NLR could result from an elevated neutrophil count, as is commonly observed in the setting of acute infectious processes, and/or a reduced lymphocyte count, as has been described previously in the setting of severe TB disease [35]. Similarly, an elevated PLR may result from reactive thrombocytosis (e.g.*,* due to inflammation) and/or a reduced lymphocyte count. None of these laboratory abnormalities are classically associated with asymptomatic TB infection, but are well-characterized in the setting of active infections, such as TB disease.

A novel observation of the present study is that certain baseline red blood cell measures (namely RBC count, hemoglobin, and hematocrit), were modestly but significantly reduced between subsequent IGRA converters compared with IGRA negative controls. Furthermore, hematocrit was significantly reduced in all IGRA converter sub-groups evaluated relative to controls. Importantly, this was not a marker of overall undernutrition, a known risk factor for tuberculosis [36], since baseline BMIs did not differ between IGRA converters and IGRA negatives (*p* = 0.91, data not shown). The reduced red cell indices observed could either represent a marker of susceptibility or a consequence of new infection prior to IGRA positivity. Anemia at the time of TB disease diagnosis is a well-documented phenomenon in the literature, most likely representing a form of anemia of chronic disease (with or without concomitant iron-deficiency anemia) [37]. Iron dysregulation has been shown to increase susceptibility to the development of TB disease among HIV-positive individuals [38], although this has not been studied in the broader population and comparable data are not available for asymptomatic TB infection. Based on prior findings in the context of TB disease as well as our own findings in asymptomatic TB infection, we speculate that iron dysregulation may increase susceptibility to the development of asymptomatic TB infection, potentially account for our finding of decreased RBC count, hemoglobin, and hematocrit in subsequent IGRA converters.

There are three limitations of the present study that should be acknowledged. First, the diagnosis of TB infection is an inherently challenging task, and IGRA itself represents an imperfect diagnostic assay. Thus, the “gold standard” against which we are evaluating the performance of MLR, NLR, and PLR, as well as other CBC measures, may have misclassified a subset of IGRA converters and/or controls. The “true” performance of these laboratory values with respect to differentiating between these two groups is therefore impossible to determine with our current diagnostic capabilities. Secondly, only a small number of study participants (*n* = 4) in the DAR-PIAT trial developed active TB disease during the follow-up period, and we consequently do not have the statistical power to compare CBC measures or CBC-derived ratios between TB-infected participants who developed TB disease versus those who did not. The study may have additionally been underpowered to detect differences in MLR, NLR, and PLR between IGRA-negative and IGRA-positive individuals given the relatively small number of participants who converted during the follow-up period. Third, because MLR, NLR, and PLR are hypothesized to represent non-specific markers of inflammation, inclusion of other controls groups, such as individuals with other chronic infections (e.g.*,* HIV) or chronic inflammatory conditions, would have been potentially beneficial. However, our study was designed primarily as a TB vaccine trial and therefore did not include such groups, although others have considered similar controls in prior studies [18–20, 23, 24].

## Conclusions

In summary, the present study demonstrates that among subsequent IGRA converters, RBC components (RBC count, hemoglobin, and hematocrit) were significantly decreased at baseline relative to IGRA-negative controls, suggesting either that impairment in RBC production may predispose to the development of subsequent TB infection or that TB infection may decrease RBC components prior to incident IGRA positivity. Of interest, despite prior studies relating MLR, NLR, and PLR with the development of TB disease, we did not find any associations between these CBC-derived ratios (or WBC components of the CBC) and asymptomatic TB infection. This comparison of TB-infected individuals with healthy controls has historically proven challenging, as TB infection does not uniformly present with signs and symptoms of TB disease and may be difficult to reliably identify given the dynamic host immune response to *M. tuberculosis*. Given the challenges associated with diagnosing TB infection, it is hardly surprising that the white blood cell components of the CBC and their associated ratio would be similar between individuals with asymptomatic TB infection and otherwise healthy controls. We conclude, therefore, that while MLR, NLR, and PLR may have utility in either the prediction of subsequent TB infection or the identification of individuals with incident symptomatic TB disease, their value in the identification of individuals with asymptomatic TB infection is questionable.

## Data Availability

The datasets used and/or analysed during the current study are available from the corresponding author on reasonable request.

## Abbreviations

AUROC: Area under the receiver operating characteristic curve
CBC: Complete blood count
IGRA: Interferon-gamma release assay
MCH: Mean corpuscular hemoglobin
MCHC: Mean corpuscular hemoglobin concentration
MCV: Mean corpuscular volume
MLR: Monocyte-to-lymphocyte ratio
NLR: Neutrophil-to-lymphocyte ratio
PLR: Platelet-to-lymphocyte ratio
POI: Prevention of infection
RBC: Red blood cell
RDW: Red cell distribution width
ROC: Receiver operating characteristic
